# Forensic Facial Comparison: Current Status, Limitations, and Future Directions

**DOI:** 10.3390/biology10121269

**Published:** 2021-12-03

**Authors:** Nicholas Bacci, Joshua G. Davimes, Maryna Steyn, Nanette Briers

**Affiliations:** Human Variation and Identification Research Unit, School of Anatomical Sciences, Faculty of Health Sciences, University of the Witwatersrand, Johannesburg 2193, South Africa; joshua.davimes@wits.ac.za (J.G.D.); maryna.steyn@wits.ac.za (M.S.); nanette.briers@wits.ac.za (N.B.)

**Keywords:** human identification, facial identification, CCTV, photography, forensic facial comparison, morphological analysis, FISWG, face mapping, disguises

## Abstract

**Simple Summary:**

Facial identification is an emerging field in forensic anthropology, largely due to the rise in closed circuit television presence worldwide, yet there is little published research in it. Our research group has conducted a series of studies testing the validity and reliability of the facial identification practice of morphological analysis. In this paper, we summarize the results of our studies and other latest advances in facial identification practice. In addition, we present a review of relevant technical literature on the limiting factors imposed on facial identification by closed circuit television, while making recommendations for practice and the future of this research niche based on a combination of our results and the technical know-how available. Facial identification research is a multidisciplinary task, with involvement from the field of anatomy, forensic anthropology, photography, image science, and psychology, among others. The value of this brief review is the bridging of these multiple disciplines to discuss the relevant needs and requirements of facial identification in forensic practice and future research.

**Abstract:**

Global escalation of crime has necessitated the use of digital imagery to aid the identification of perpetrators. Forensic facial comparison (FFC) is increasingly employed, often relying on poor-quality images. In the absence of standardized criteria, especially in terms of video recordings, verification of the methodology is needed. This paper addresses aspects of FFC, discussing relevant terminology, investigating the validity and reliability of the FISWG morphological feature list using a new South African database, and advising on standards for CCTV equipment. Suboptimal conditions, including poor resolution, unfavorable angle of incidence, color, and lighting, affected the accuracy of FFC. Morphological analysis of photographs, standard CCTV, and eye-level CCTV showed improved performance in a strict iteration analysis, but not when using analogue CCTV images. Therefore, both strict and lenient iterations should be conducted, but FFC must be abandoned when a strict iteration performs worse than a lenient one. This threshold ought to be applied to the specific CCTV equipment to determine its utility. Chance-corrected accuracy was the most representative measure of accuracy, as opposed to the commonly used hit rate. While the use of automated systems is increasing, trained human observer-based morphological analysis, using the FISWG feature list and an Analysis, Comparison, Evaluation, and Verification (ACE-V) approach, should be the primary method of facial comparison.

## 1. Introduction

Cameras and photographic imagery have been used in surveillance, identification, and detection of criminals as early as the 19th century [1]. Anthropological standards have been used to depict portraits of regular criminals for law enforcement registries, similar to today’s mugshot system. These registries were intended as a means for witnesses and victims to conduct a facial review of potential suspects. However, the lack of standardization in image capture processes made these registries ineffective. The advent of judicial photography, in the late 19th century, incorporated anthropometry and relied on standardized conditions of image capture, featuring the well-known anterior and lateral facial views with neutral expression and stance [1,2] routinely used to this day by many police departments throughout the world. The facial anthropometry application was abandoned in favor of the more accepted fingerprint identification system [3], yet the facial image capture standards it relied on endured in facial depiction practices throughout the 20th century [1].

Depicting faces [1], facial anthropometry [2], and facilitating crime scene investigations [4,5] have relied on the use of photography in a forensic context almost since its development [1]. Probably the most recognized use of photography in a forensic setting, and its derivative in the form of video recording, is surveillance. Closed-circuit television (CCTV) was the natural progression of improved use of video technology that allowed for consistent monitoring and review of potential criminal activities [6]. CCTV surveillance systems have since the 1990s become increasingly more common and relied upon throughout the world [7,8,9,10] and are in fact considered by many communities the norm in public areas [11,12].

Deployment of CCTV surveillance is considered to act as a deterrent for local crime in monitored areas [8,13,14], often shifting criminal incidents to nearby unmonitored areas instead of completely eliminating them [10]. However, perhaps its most valuable contribution is its frequent use in criminal investigations [8,15]. An analysis of CCTV data in the United Kingdom showed that when CCTV data are available, criminal activity is substantially more likely to be resolved [15]. When the data were not of use, it was primarily due to its lack of availability or some fixed parameter of the surveillance system being suboptimal, such as the incident not being covered by CCTV, the system being faulty, or the images being of insufficient quality [15]. The criteria of usefulness of CCTV recordings vary greatly based on the intended use.

Other than general surveillance and criminal activity monitoring, facial examination is often of interest for the data extracted from many CCTV surveillance systems. This has become more evident as the deployment of CCTV systems and increases in crime have led to an increase in demand for facial identification [16,17,18]. This rise in demand is a direct outcome of the increased availability of image data, from both CCTV data [7,16] and photographic and video evidence from other sources, such as mobile phones [19].

Forensic facial identification falls under the discipline of facial imaging, which involves the use of visual facial data to assist the identification process [20]. Through the analysis of photographic or video evidence, forensic facial identification is routinely utilized to associate persons of interest to criminal activity [17]. Craniofacial identification involves multiple disciplines, such as facial approximation, facial composites and sketches, age progression and regression, photographic superimposition, molecular photofitting, facial depiction, and facial comparison [20]. Some of these techniques, such as facial approximation and facial composites and sketches, have been researched in some depth [20]. However, forensic facial comparison (FFC) for identification remains largely untested, despite its increasing demand [17,21].

Understanding that forensic facial comparison is a niche of research that needs further development requires the use of clear terminology. A colloquial confusion in terminology between facial identification and recognition is prominent throughout many discussions. This misnomer has been discussed by Schüler and Obertová [22], who clarified that identification is reliant on perfect agreement, which is different from recognition, understood as the innate psychological process humans employ at a glance to recognize a face, usually based on familiarity. Therefore, to attempt facial identification from a forensic anthropological perspective, a strict process of facial comparison is employed. Due to the innate process of recognition in any forensic facial comparison process, the distinction needs to be made clear. Recognition is employed generally as part of the investigative process of facial comparison and is holistic, rapid, and methodologically inconsistent with a high predisposition to error [23,24]. Identification, however, requires further systematic analysis involving standardized, detailed, comprehensive, and meticulously recorded methodology [22]. As such, forensic facial comparison must involve the human-based detailed examination of facial images for identity confirmation [25,26,27].

Another prominent misconception in facial identification (ID) involves the misuse of the term “facial recognition” to specifically refer to automated or semi-automated facial recognition systems, with this being fully adopted by many in the field of automated facial recognition (e.g., [28,29]). To avoid this miscommunication, certain studies refer to automated facial recognition as facial recognition technology (FRT) or systems [30]; however, this practice is not universally applied.

The misnomer of FRT and facial ID is often closely associated to the misconception of FRT being considered the ideal approach to facial ID. FRT systems apply a variety of computer-based methods to attempt confirmation of facial identity [29,31] and have proven high levels of accuracy in constrained circumstances [28,29]. While great advances have been achieved in the field of FRT [28,32], it remains associated with high false positive rates [32,33], strong racial biases [34], and other ethical concerns around privacy and consent that require resolution prior to the employment of FRT in a legal context. Most concerns revolve around the reliance of FRT systems on biometric information [35] and highly standardized images [36,37,38], which are often not available in the realistic unstandardized organization of most surveillance installations. As a result, while there are strong commercial and government incentives to deploy FRT systems, in part due to their large market share (USD 3.72 billion) [39], they are still reliant on human-based validation in their operating loops [40]. The need for human validation is further enhanced by the lack of varied databases used to develop and test these FRT systems [41]. Hence, until further varied and realistic databases are used to test and develop these FRTs, human observer-based facial image comparison is considered the preferred approach to facial ID [25,42,43,44] and will likely persist as the validation method of choice despite the improvement and widespread deployment of FRT systems.

Understanding the limitations and permissible applications of FRTs is crucial to conducting research in both FFC and FRT. The misconceptions and assumptions around FRT and FFC may pose a risk of driving researchers and funders away from conducting research in facial identification. This is primarily because most funders and new researchers would consider facial identification, and particularly FFC, as redundant in an era where FRT has become the norm. Despite these misconceptions, human-based facial identification methods, which are currently employed routinely in the judicial system, rely on forensic facial comparison [17,42].

Facial examination, also referred to as forensic facial comparison (FFC), must be applied using the Analysis, Comparison, Evaluation, and Verification (ACE-V) approach [27], commonly used in other forensic practices, such as fingerprint identification [45]. The ACE-V methodological approach is meant to integrate principles of the scientific method in forensic comparisons in order to enhance their implementation and reliability [45].

**Figure 1:**
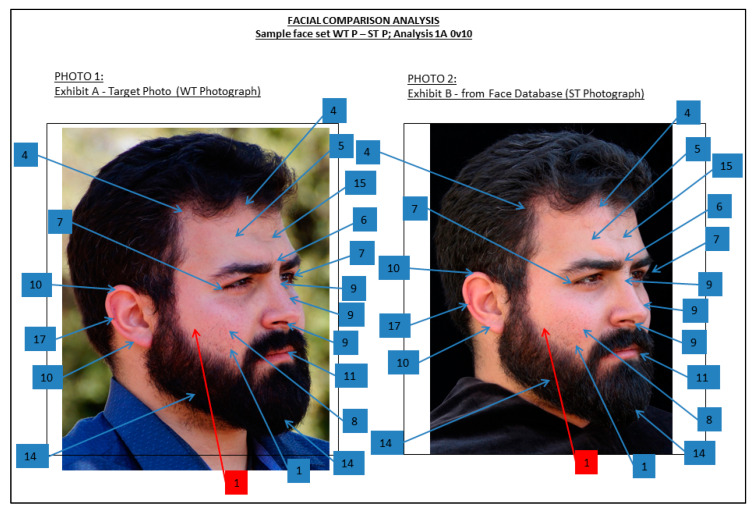
Example of a forensic facial comparison analysis process between a wildtype (WT) photograph and a standardized (ST) photograph from the Wits Face Database [41] sample images in the SAPS court chart format. The individual facial features are numbered, analyzed, compared, and evaluated between the two images using the FISWG feature list [47]. Features marked in blue indicate morphological similarity between the two images, while features marked in red indicate morphological dissimilarity. In the example provided, skin color appears different due to lighting discrepancies in the two images (red 1); however, skin texture appears similar (blue 1). The facial images used for Figure 1 are images of the corresponding author of the present manuscript and are part of the sample images of the Wits Face Database [41], reproducible under an open access license distributed under the terms of the Creative Commons Attribution License. This license permits unrestricted use, distribution, and reproduction in any medium, provided the original work is properly cited. The images can be found in the Wits Face Database data note, including the supplementary material for the Wits Face Database [41].

In the past, approaches to FFC included photo-anthropometry, facial superimposition, and morphological analysis (MA) [20,27], with morphological analysis being the currently accepted method as advised by both the Facial Identification Scientific Working Group (FISWG) (https://fiswg.org/index.htm accessed on 30 October 2021) and the European Network of Forensic Science Institutes (ENFSI) (https://enfsi.eu/ accessed on 30 October 2021) [27,46]. Application of MA relies on the detailed examination of specific facial features to reach a conclusion with regard to the similarity or dissimilarity of two or more faces [27]. The facial features are assessed subjectively, evaluated, and compared between the faces [27]. The selection of individual facial features often depends on the feature list utilized. Feature lists generally include both overall face composition and structure, individual anatomical feature components (e.g., hairline shape, ear helix morphology, nasal alae protrusion, etc.), and distinguishing characteristics such as scars, blemishes, piercings, and tattoos (e.g., [47]). The current standard feature list used for facial comparison relies on criteria developed by the FISWG for facial comparison by MA [47]. An example of how this analysis is conducted is shown in Figure 1, using sample facial images from the Wits Face Database [41].

Recently, our research group (https://www.wits.ac.za/anatomicalsciences/hviru/ accessed on 30 October 2021) has conducted a series of validation studies to test the validity and reliability of FFC using the FISWG list (https://fiswg.org/index.htm accessed on 30 October 2021) of morphological features [21,41,48,49]. The aim of this paper is to summarize the results of these findings, thus elucidating the reliability and potential uses of FFC. Potential areas of caution and observed shortcomings are also discussed. Finally, recommendations as to the minimum standards for CCTV equipment are given, as well as guidelines for future directions in research.

## 2. Development of an African Facial Image Database

Although various facial databases exist (e.g., [50,51,52,53,54,55,56,57,58,59,60,61]), none of these were suitable for the systematic and blind testing envisaged for the purposes of the current stream of research on FFC validation. Some of these databases have small numbers of faces (e.g., [62,63]) or contain low-resolution images (e.g., [51,64]). As these databases were developed with different purposes in mind [65], and, with the exception of one [55], do not contain African faces, a new database was needed. A database containing African faces would also be invaluable in future research on the African continent.

Such a database was developed for the purposes of these studies, but due to the magnitude of such an undertaking, currently only males are included. This new Wits Face Database includes a total of 622 unique African male individuals aged between 18 and 35 at the time of recording, each with 10 photos associated to them, in five different views (anterior, left and right lateral, and left and right 45°) [41]. The 10 photographs were captured with high-resolution midrange cameras across two different conditions: a controlled setting with uniform background and obscured clothing at a subject-to-camera distance (SCD) of 1.5 m and an uncontrolled setting with a mixed background and visible clothing at an SCD of 5 m. This brought the total to 6220 facial photographs [41]. Out of the 622 participants, 337 (54.2%) were also recorded under different CCTV recording conditions [41]. The first group, recorded under a standard digital IP CCTV installation at approximately 3 m height, included 89 individuals; the second group, recorded at an eye-level digital IP installation (1.7 m installation height), included 76 participants; the third group included 107 participants, recorded by an older analogue CCTV installation (2.5 m height); and the last group, recorded by the same digital IP CCTV camera as the first group, included 34 and 31 participants wearing caps and sunglasses, respectively [41]. Throughout the CCTV data, large amounts of data loss were experienced, particularly with the internet protocol (IP) CCTV cameras, due to corruption, compression, and intermittent connectivity (Table 1).

While the inclusion of males only is a good step towards expanding the diversity in populations included in face databases, the non-existence of a female database remains a notable limitation to be aware of. In principle, the FISWG feature list should be generic enough to make it applicable across sex and population groups, but facial variations may potentially lead to variations in accuracies and reliability based on the biases and abilities of the observers. The existence of a within-group face recognition advantage (previously called own- or cross-race bias) has been well described and may play a role in the reported accuracies of FFC [66,67,68,69,70,71]. It is, therefore, essential that future databases include faces that are representative of all major populations. The newly developed database is now the largest African database of CCTV recordings and matching high-resolution facial photographs. It is available for all bona fide research that meets the criteria as set out by the Human Research Ethics Committee (HREC) (Medical) of the University of the Witwatersrand [41,72].

## 3. Outcomes of Validation Studies

**Figure 2:**
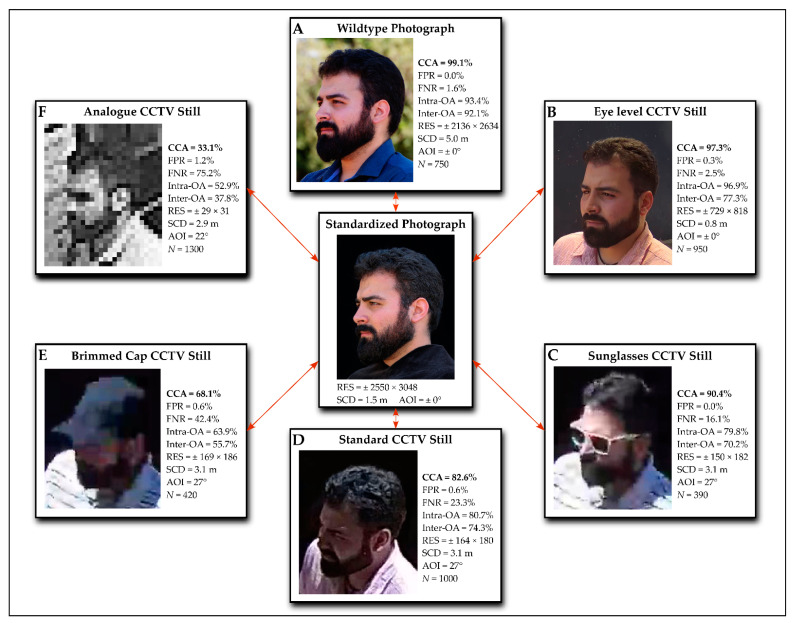
Visual summary of the validation studies testing morphological analysis across realistic photographic and CCTV conditions [21,48,49] using sample photographs and CCTV stills from the Wits Face Database [41]. Images (**A**) to (**F**) are samples of the target images from each set of conditions analyzed that were compared to the central image arising from the standardized photographs captured for each participant. All major statistical results and the details of the conditions of each comparison cohort are presented. Representative images of each condition are arranged from A to F in a clockwise order according to descending chance-corrected accuracy. The conditions of analysis were as follows: wildtype informal photographs (**A**) of similar quality to the standardized photographs; eye level digital CCTV still images (**B**); standard digital CCTV still images (**D**) with sunglasses (**C**) and with brimmed caps (**E**); and monochrome analogue CCTV still images (**F**). Key: CCA = chance corrected accuracy; FPR = false positive rate; FNR = false negative rate; OA = observer agreement; RES = resolution; SCD = subject-to-camera distance; AOI = angle of incidence; *N* = number of comparisons. The facial images used for Figure 2 are images of the corresponding author of the present manuscript and are part of the sample images of the Wits Face Database [41], reproducible under an open access license distributed under the terms of the Creative Commons Attribution License. This license permits unrestricted use, distribution, and reproduction in any medium, provided the original work is properly cited. The images can be found in the Wits Face Database data note, including the supplementary material for the Wits Face Database [41].

Various standards exist worldwide as to how to express the levels of confidence when it comes to possible matches. In Australia, for example, facial comparison experts are expected to present evidence strictly in descriptive terms, which can lead to suggestive language, based on the expert’s prejudice and opinion [26]. In England and Wales, FFC experts report on comparisons based on the Bromby scale of support [73], where the scales of support force experts to conclude whether two compared faces are a match regardless of image conditions or quality [26]. The Bromby scale is also inherently arbitrary with no clear distinction between each step of the scale. To alleviate these uncertainties, experts from the South African Police Services (SAPS) make use of a five-point scale that reflects the ability of an expert to analyze a given set of images, as well as the confidence level of a specific conclusion [17]. For application and testing, this scale was slightly adjusted to allow statistical testing to reflect an order of severity of conclusion. Namely, a score of 1 was assigned to confident positive identifications, a score of 2 to inconclusive identifications that showed some level of morphological similarity on certain specific facial features, a score of 3 that represented an inconclusive identification with overall holistic similarity of two faces compared, a score of 4 as a negative identification, and a score of 5 indicating impossible to analyze due to insufficient visibility of landmarks [21]. A visual overview of these outcomes is shown in Figure 2.

Morphological analysis on data derived from the newly developed Wits Face Database [41,72] using the FISWG feature list [47] was found highly accurate and reliable when comparing optimal standardized photographs to wildtype (informal) unstandardized photographs [21]. In an analysis of 75 sets of faces (each containing nine no match comparisons and one positive match comparison or 10 no match comparisons—compared to a target image, total *n* = 750 comparisons), the chance corrected accuracy and reliability were found to be almost perfect in optimal photographs (99.1% and 92.1%, respectively) [21]. In the analysis of 100 face sets (*n* = 1000 comparisons) with standard digital CCTV recordings as the target image, a lower accuracy (82.6%) and reliability (74.3%) were noted [21] (Figure 2). The lower performance of MA in standard CCTV was ascribed to the variation of conditions of the different equipment and its installation. Specifically, images obtained from the standard CCTV system were of poorer quality than the high-resolution controlled and wildtype photographs, due to a number of reasons. Firstly, the image resolution of the standard digital CCTV camera was lower (4MP) than that of the photographic cameras (18MP) [21]. Secondly, the CCTV field of view was broader and less focused on the face, partly due to the SCD being approximately 3 m. As such, a larger area was captured at a lower resolution, effectively reducing the actual resolution of the recorded faces [21]. Thirdly, between the CCTV camera and the captured face, an angle of incidence of 27° was formed, which appeared to limit visibility of the face, potentially shifting relative proportions of facial features [21]. The change in perspective and the limitations it placed on the facial comparison process likely contributed to the lower accuracy and reliability seen in the standard CCTV conditions [21].

Image lighting was also markedly different between photographs and CCTV recordings, making facial characteristics reliant on color (i.e., skin tone, luminescence, and color) redundant, since they appeared different even between matching images [21]. Variations in lighting also contributed to over-exposure of certain features, effectively limiting their utility in facial comparison [21]. Beyond these discrepancies and concerns, the almost perfect accuracies and the low false positive rates identified (<1.6%) (Figure 2) are encouraging for the use of MA in a legal context from both optimal photographs and standard CCTV installations [21].

Following on the first set of analyses under fairly optimal conditions, a second set of tests was done on 130 face sets (*n* = 1300 comparisons), arranged as described above, recorded on a low-resolution suboptimal analogue CCTV system. The results were found to be much poorer, with accuracies as low as 33.1% with extremely high false negative rates (75.2%) and questionable reliability (37.8%) [48] (Figure 2). The contributing factors to this decrease in accuracy were a pronounced angle of incidence (22°), lack of color, and particularly the low-resolution images [48]. However, determining which of these specific factors contributed the most to the low accuracy is not possible by study design, but the decreased quality of the images seems to be the most problematic factor [48]. The contribution of lacking color, however, is questionable, as facial examiners in certain countries conduct their comparisons in greyscale with the consideration that color can be considered misleading. This effect of color was also observed in a previous study, where attempting to match skin color between images proved futile due to lightning discrepancies between images [21]. Irrespective of the specific contribution, the combination of these factors was highly disruptive to the facial comparison analysis—even more so than the inclusion of disguises [49].

The above suboptimal comparisons were contrasted to 95 face sets (*n* = 950 comparisons) recorded at eye-level with a digital IP CCTV camera. As can be expected, eye-level digital CCTV images were found to yield better results than the standard CCTV installation [21,48]. An effective 0° angle of incidence and a much smaller SCD of 0.8 m seem to have simulated the most ideal CCTV conditions for facial comparison [48]. In fact, eye-level digital CCTV recording-based facial comparisons were almost as accurate (97.3%) and reliable (77.3%) as the standardized photograph to unstandardized photograph comparisons [21,48] (Figure 2). This outcome is telling of the factors that may have played the biggest role being angle of incidence and SCD, since the standard CCTV and the eye-level CCTV were identical cameras installed at different conditions [21,48]. However, to assess the extent of the influence these factors had on facial comparison, further targeted testing of these individual factors is required.

During the analysis of the data from the facial comparisons, two iterations were conducted—the strict and the lenient iterations. Under a strict iteration, only a confident positive identification was taken as a match, while under the lenient iteration, even inconclusive analyses with some morphological similarity in facial features were considered as matches along with the positive identification [17,21]. When reviewing the performance of MA in the analogue CCTV data, it was noted that a significantly altered performance resulted under different levels of analysis strictness. A strict iteration resulted in a worse performance in the analogue CCTV comparisons than across all other comparisons (photographs, standard CCTV, and eye-level CCTV) [48]. All other analyses from the various CCTV and photographic images showed improved performance under a strict iteration [21,48,49]. This outcome advocates that under particularly suboptimal conditions, such as analogue CCTV, even a strict approach to the analysis is ineffective in improving performance. However, the decreased accuracy under a strict iteration may be worth considering as a marker of suboptimal conditions. Effectively, when a strict iteration results in lower performance of MA in a particular dataset than a lenient iteration, that dataset should be viewed as being below a usable threshold for facial comparison. As such, recordings that perform worse in a strict iteration, particularly in cases where target exclusion is not possible, should be avoided for positive identification. Effectively, when testing the performance of MA in a given dataset extracted from a specific CCTV installation, both a strict and lenient iteration should be conducted. Should the strict iteration perform worse than the lenient iteration, then the specific CCTV installation that yielded that footage should be considered below a usable threshold for the purpose of FFC. This consideration of statistical analyses is included in our recommendations on how to conduct MA.

Across all of our studies, the best measure of accuracy was found to be the chance corrected accuracy (CCA) [21,48,49]. CCA was calculated by conducting a weighted Cohen’s kappa (with squared weighting) on the assigned scores for each comparison contrasted to the actual true match-up information for each comparison trial. This is different to the normal hit rate or raw accuracy, which simply indicates the amount of correctly scored trials irrespective of the degree of error or the sample composition. This is also different to the balanced accuracy that is calculated when computing a confusion matrix analysis, which is effectively the sum of the sensitivity and specificity divided by two [74]. In the studies’ results, CCA varied the most and was seen as the most representative measure of accuracy, particularly when compared to the simple hit rate and balanced accuracy. These two accuracies presented skewed results towards true negatives due to the studies being conducted under a one-to-many comparisons context, an approach to facial analysis also seen as the harshest testing criteria for automated facial recognition systems [75,76]. As a result, the non-chance corrected accuracies appeared deceptively higher due to the high prevalence of true negative matches, despite other measures of performance indicating a more questionable outcome. With this consideration, future FFC studies should consider making use of CCA as their primary measure of accuracy as opposed to hit rate, historically the most common measure of accuracy.

Beyond the optimization of surveillance system installations specifically for facial comparison, an additional limiting factor investigated in these studies were the effects of disguises on facial comparison. We specifically investigated the effect of sunglasses (*n* = 390) and brimmed caps (*n* = 420) on FFC performance [49] (Figure 2). Overall, the performance of MA in faces disguised with sunglasses was markedly high (90.4%) [49], in fact surpassing the performance of facial comparison under the same standard CCTV conditions without sunglasses (82.6%), but not better than the photographic (99.1%) [21] or eye-level CCTV data (97.3%) [48] (Figure 2). This unusual consequence of sunglasses on facial comparison has also been observed by Davis and Valentine, who tested live subject to image identification [77]. These authors [77] suggested that the instruction that was given to participants conducting face matching tasks to rely on the external facial features with subjects disguised by sunglasses apparently increased their ability to recognize a face disguised by sunglasses. External facial features are in fact considered the most reliable set of features in unfamiliar face matching, as corroborated by other studies [78,79]. In FFC, conducted using the FISWG feature list, a methodical approach with a focus on all facial features including the external ones was followed. As a result, this methodical approach may have indirectly contributed to avoiding the limitation that sunglasses would normally pose on this comparison cohort. In contrast, faces disguised by brimmed caps yielded an exceedingly low CCA (68.1%) [49], yet not nearly as low as the analogue CCTV comparisons (33.1%) [48] (Figure 2). The limitations posed by brimmed caps appeared to have been compounded by the large angle of incidence of the standard CCTV recordings as well as the strong natural lighting from the sun. These two conditions, in conjunction with the brimmed caps, created shadows over the face, obscuring an even greater number of facial features, resulting in large-scale information loss [49]. This effectively rendered comparison much more difficult, as less than the lower half of the face and the ears could be evaluated [49].

Eyeglasses and various types of hats have historically been viewed as the most inconspicuous and common disguises [25,80,81]. Although the specific effects of various disguises have been discussed broadly, only one study has attempted applying MA to a disguised sample [49]. Despite their lack of testing in MA, in face matching recognition, brimmed caps were found to increase error rates over other comparison tasks [81]. Brimless caps and glasses, on the other hand, appear to have a less pronounced effect on match accuracy, varying by the method employed [82,83].

The success of MA in disguised faces was credited in large part to the FISWG feature list [47]. The use of even rudimentary feature instructions or even partial feature lists is able to increase the performance of facial comparison analyses [84,85,86], with a more pronounced effect noted for trained experts [84]. Our results from the disguised test of MA [49] reinforce these outcomes of other studies, further supporting the use of feature lists in MA.

## 4. Discussion

This paper summarized the outcomes of our recent studies testing MA and the FISWG feature list across varied conditions of facial CCTV images and photographs. In addition, it presented and discussed the major limitations of FFC. MA of faces, using a feature list, is accurate and valid, particularly when conditions are optimal (e.g., high-resolution photographs and high-resolution CCTV with limited perspective distortion/angle of incidence). Image quality had the most notable effect on facial comparison performance (analogue CCTV recordings), while brimmed caps were found to be the second-most limiting condition. Across both of these conditions, the major limiting factor appeared to be overall loss of facial feature information, with caps obscuring almost half of the face and the poor quality of analogue video material making most of the facial details indistinguishable.

### 4.1. Influence of CCTV Installations

To determine the minimum criteria for facial examination across various CCTV installations, a more thorough understanding of the conditions imposed on footage by specific installations is needed. This is of particular relevance with the continuing global increase in the installation and usage of CCTV systems that has been seen across private, public, and commercial sectors in the last two decades [7,46,87,88]. This increase can be attributed to multiple factors; however, two major drivers include advancements in computing and CCTV system production and a reduction in the associated costs [7]. The vast global increase in CCTV deployment has led directly to an increase in available data for use in potential criminal surveillance and related investigations.

While this global increase in CCTV data is beneficial to criminal investigation and facial comparison, there is a concerning lack of standardization of required installation, recording conditions, and image quality [20,82,89,90,91,92]. As a result, the usefulness of CCTV-derived facial images is difficult to assess and makes facial comparison challenging in contrast to controlled photographs and mugshots. These limitations along the CCTV imaging chain are often acknowledged; however, few studies have assessed their implication in facial comparison accuracies [21,48,82,92,93,94]. Successful facial identification assessment is hindered by inconsistent recording conditions and poor image quality. Facial comparison accuracy and data quality are, thus, directly correlated [95,96], especially in terms of individual accuracy variation across multiple analysts [97] and individual analyst ability overestimation [98].

CCTV camera placement is one of the major limitations in terms of recording conditions. Most surveillance systems are put in place in order to monitor large crowds or entry/access points and do not have FFC in mind. The placement of the camera is based on the field of view that can be monitored and is then complemented by the mounted height above ground. Camera height relative to subject distance gives the angle of incidence, and this is an important, and often detrimental, component for extracting facial details from recordings.

Typical surveillance camera mount heights are between 2.5 and 3 m on building exteriors and ceiling height for indoor surveillance [99]. The main justification behind these mounting heights is that it lowers the risk of cameras being vandalized, stolen, or obstructed. The problem with these standardized mounting heights is that they translate to a steep angle of incidence. This in turn reduces image quality and obscures relevant facial detail as a result of the increased SCD and subsequent loss of useable resolution [48,49]. This is particularly important in facial comparison, as the amount of visible facial features and the view in which the face is seen are crucial for successful identification [100]. People also tend to naturally tilt their heads inferiorly by 15–20° when walking [100], thereby further exacerbating this problem. The current recommended angle of incidence limit is 15°, as any steeper angle would result in significant loss of facial detail [100]. Eye-level mounted cameras at 1.8 m ground height provide an approximate 0–15° angle of incidence with the subject and provide the most optimal capture of facial detail even with natural head tilt [48]. Further individual variations in facial view, or pose, are additional factors that may further affect facial comparison, even at eye-level CCTV placement, particularly under poor quality and capture conditions [48,101,102]. Our reports [48] show an overall better and more reliable performance of MA in a digitally captured sample (IP cameras) at eye-level height (1.7 m) compared with a suboptimal sample at an angle of incidence of 27° (mount height of 3.1 m) [48]. In contrast, our analogue CCTV data, captured at an angle of incidence of 22° and height of 2.5 m, performed worse overall, although it is unclear if this was because of camera position or poor image quality [48].

Camera placement, particularly in relation to positioned angle and mounted height, dictates the monitoring area, while the camera lens, its focal length, as well as the sensor, specifically its size and number of pixels, dictate the field of view, image quality, level of optical distortion, and noise present [99]. The distance between the camera and a subject or target will then affect the image composition, which directly affects target size on sensor or picture height, and level of perspective distortion [92,103,104]. All of these factors and components will affect the usefulness of an image for facial detection and subsequent comparison analysis. Monitoring a large crowd outside a building, for example, requires 5% of picture height, while detecting a specific target requires 10% [99]. A potential target must occupy more than 400% of screen height in order to conduct facial examination, and a minimum of 1 mm must be represented per pixel of the whole image (ISO62676 recommendations) [99]. Considering the conservative European standards for facial image comparison [105], a minimum of the top quarter of a subject must be included on screen height and the face would need to represent a minimum of 1000 pixels per meter of screen height [99]. As such, for each inch (2.5 cm) of a face represented in an image, a minimum of 25.4 pixels is required [99]. For this minimum pixel density to be maintained at set SCDs, certain lens focal lengths need to be utilized. For example, at a 5 m distance from camera to subject, a focal length of 4.2 mm is necessary on a ½” sensor HD CCTV camera [99], which is considered a common IP camera type. The longer the focal length of the lens, the narrower the field of view; simultaneously, the smaller the camera sensor, the smaller the viewing angle and the higher the noise. Bigger sensors and higher pixel counts are, in theory, always better for security and forensic applications, especially in low-light performance; however, bigger optics are then also required, which increases camera size, weight, power, and most importantly, cost.

Lighting conditions can pose further challenges in recording optimal footage. Facial details may be lost to over- or under-exposure of a subject and may not be retrievable through post-processing [99]. In outdoor locations, the position of the sun and related shadows, the amount of ambient lighting based on time of day, or the combination of multiple light sources or reflective materials near the subject or camera all could lead to unbalanced exposure. This then ties to the sensitivity of the camera sensor and its dynamic range capability. Most modern IP cameras are better suited to handling high-contrast environments, but older analogue systems generally provide either over- or under-exposed coverage with limited middle grounds [99]. Harsh and high-contrast lighting conditions often create artificial boundaries on viewed objects, altering appearances and reducing the accuracy of facial identification [99]. Over- and under-exposed footage may render an analysis impossible, based on multiple facial features being completely unrecognizable [21,106].

The capability of the camera is the primary factor in terms of low light or night-time conditions. Without the addition of directed lighting or dedicated “night-vision” cameras, CCTV systems must incorporate cameras that can record with infrared radiation (IR) and convert to visible light [107,108]. The accuracy of FFC has not been tested under IR conditions in our recent work and remains to be done in future studies. Most modern analogue and IP cameras are able to switch between day/night recording automatically and have IR LEDs built in to illuminate the target area. The range of the IR is generally limited to 20 m for midrange cameras on the market. This IR source of light could itself over-expose the subject dependent on SCD and other reflective materials present [99]. In addition, the IR footage is recorded in monochrome, and therefore, includes the same limitations and challenges of traditional black and white CCTV footage in facial comparison [93], although in the current work, color was found to not be of much importance. Lens distortion effects and optical aberrations are more pronounced in IR cameras because of the longer wavelengths of IR [108]. Lastly, IR recording is subjected to image alterations and other artefacts based on converter quality and functioning [108]. Experimentally, IR recordings are difficult to conduct with subjects, as recording conditions need to be in low-to-zero light levels. There is a significant lack of research on IR CCTV recording in the context of facial identification and testing, and further validation of MA on a sample of IR surveillance data of comparable quality and conditions to the standard CCTV camera should be conducted.

As discussed above, placement and recording conditions of CCTV systems are crucial for reliable data capturing and use, especially in a forensic evidence context. This is inclusive of its installation in terms of network, software, and hardware. Many complications can arise as a result of these factors. Some examples experienced when attempting to develop the Wits Face Database [41,72] included inconsistent IP network connection and coverage, power outages, imminent weather problems, theft, and finally data loss, corruption, and tampering. Analogue CCTV systems for the most part do not provide remote video access and therefore require a physical storage and viewing location, limiting flexibility. These systems by default record at lower resolutions and require immediate local storage on a DVR device. This generally translates to a reduced amount of data loss and corruption compared to digital systems. Digital video can be recorded with varying rates of resolution, frame rate, and levels of compression [90]. The linkage of digital IP cameras to the internet allows for transmission of recorded footage for remote viewing, which requires high processing, storage, and data transmission capabilities. Digital video is, thus, more prone to occasional partial or complete data corruption or loss and is more perceptible to anti-forensic techniques, such as removing, hiding, and corrupting or wiping evidence from recorded footage [109,110,111,112]. In light of these threats, forensic readiness is needed in modern CCTV systems from both physical and cyber-attacks.

Little data exist describing the types and quantity of data loss incurred in CCTV systems globally and how this impacts surveillance and criminal investigations. Our studies [41] found approximately 21.2% loss of IP CCTV data and approximately 3.6% loss of analogue CCTV data during the establishment of the Wits Face Database (Table 1). CCTV data loss was noted in both IP and analogue cameras; however, the majority of corrupt or permanently lost data occurred with the digital IP camera systems. The CCTV systems utilized were an existing network at the university with no local storage and immediate transfer to a central server. During data transfer, any interruptions or fluctuations in local area network traffic or connectivity would result in data loss or irreparable corruption [41]. Studies utilizing existing CCTV systems and recordings are subjected to these types of data loss and corruption unless equipment is personally procured and installed. Data capture delays and reduced sample sizes are a considerable limitation when developing or expanding facial image databases.

The above discussed recommendations and primary limitations are generally not adhered to or considered, as is reflected in the actual data handed over to or available to law enforcement. Oftentimes, these data are of a subpar quality as a result of the numerous limitations as well as outdated camera systems [82]. Even with this subpar quality data and its limited utility, in a judiciary context, they may still successfully be implemented and should not be excluded until thoroughly reviewed first [113]. Thorough consideration of available evidence is in line with the ENFSI recommendations of triaging image data by their quality to ascertain fruitful use of FFC and efficient caseload management [114].

CCTV system installation and recording conditions are purpose driven and situationally applicable. They differ vastly to one another in terms of functionality, reliability, and environmental fit. System installation, hardware, and software need to be balanced in order to achieve the best result in terms of cost. Most systems are notably still lacking in applicability for facial comparison and are primarily disadvantaged not by installation and recording conditions but by image quality [20,82,89,90,92].

The poorer the derived image, whether it be from a photograph or CCTV footage, the lower the amount of extractable information. Image quality itself is a combination of multiple factors and related artefacts, with some of the relevant ones being resolution, pixelation, and noise. All of these are conditions that can vary notably across the various types of CCTV systems.

Analogue CCTV systems generally have lower resolutions and higher noise (grain) and often only record in monochrome. These cameras have been the global standard, and only in the last five years have we seen a large shift to internet protocol (IP) cameras [115,116]. The lower resolution leads to higher noise when attempting to enlarge the captured image for analysis and produces low clarity images [99,113].

The lack of color in most analogue recordings has a large impact in subsequent analysis, particularly in facial comparison [93]. Color plays an important role in face detection and recognition in humans, even when image quality is poor [117,118]. CCTV systems in general do not accurately capture color information from a scene [119] and have been deemed mostly unreliable in a forensic context [91,120]. Subject illumination as well as the color, orientation, and texture of objects are the primary variables dictating the accuracy of captured color information in CCTV [91]. When conducting MA using the FISWG facial feature list [47], color is the first component, and therefore, inaccurate image color data may lead to a decreased accuracy in performance. While color was easily disregarded in the majority of the analyses conducted in our studies, considering its contribution and consistency across CCTV recordings and photographs may be important for future studies.

More modern and commonly used internet protocol (IP) CCTV systems generally record full color at much higher resolutions with lower noise, as a result of high-spatial frequency blocking, overall leading to better extractable information for analysts [107]. Digital video is also a lot more flexible in recording and streaming quality compared with analogue in terms of video resolution, frame rate, and compression [90].

Poor quality CCTV recordings and extracted images have been shown to affect face matching ability in both novice and experts and leads to high overall false positive rates [82]. Image pixelation or spatial quantization, as a part of overall image quality, also drastically affects face matching ability [121]. Highly pixelated images can reduce face matching abilities by up to 50% in trained individuals when compared to a high-quality image sample [94,98]. In general, all forms of facial comparison accuracy will suffer when using low-resolution analogue CCTV images, even if image quality is good in other respects [48,81,93,122].

If we consider the SCD, the further away the subject, the greater the loss in detail in terms of representation of the face on the image. A minimum horizontal pixel count of 10–16 per face for a known face [121,123,124] and 20 pixels for an unknown face [92] is considered the bare minimum for successful identification in frontal view. Based on relative subject size on screen, Vitek et al. [125] recalculate Utochkin’s [126] recommendations to 35 pixels for a known individual and 83 pixels for an unknown individual. If we are considering the effects of pixelation in a forensic setting, one needs to address the performance of matching accuracies and any form of potential enhancement, such as image blurring and reducing image size, when viewing [92]. Another important factor along the CCTV imaging chain not discussed here is that of the display fidelity and how the image is viewed on screen and the type of screen or monitor used [91].

Subject-to-camera distance can also result in facial distortion that alters facial proportions and shapes. While not exclusively investigated in the context of MA, previous work by Stephan and colleagues has looked at the SCD induced distortion and craniofacial superimposition [103,104,127]. Stephan [103] identified that discrepancy in camera-to-face, or skull, distances between photographs to be compared, presented with varying degrees of perspective distortion of facial features. At shorter distances, particularly below 6 m, the distortion was found to be more pronounced [103]. While distance-based perspective distortion is even more important in methods applying facial morphometry since at distances below 1 m a difference of 100 mm in SCD between the compared images can result in perspective distortion greater than 1% [103]. While perspective distortion would expectedly affect morphological comparison of facial features, the qualitative approach of MA and the large number of features being compared in each analysis would likely mitigate any small degrees of perspective distortion of compared images. However, further study into the effect of perspective distortion on qualitative assessment of facial features would be necessary.

The last, but important, limitation to consider in terms of image quality is video compression. As mentioned previously, digital video quality and corresponding file size can be made smaller in three ways—decreasing frame rate (e.g., 60 fps down to 5 fps); decreasing video resolution (e.g., Common image format (CIF) to Quarter CIF); and finally, by employing video compression [90,125,128]. Software video compression manipulates the spatial and temporal redundancy of moving frames in the form of CODECs, such as MPEG-4, Wavelet, H.265/HEVC, and JPEG [90,125,128]. Compression allows for large quantities of captured data to be stored in highly reduced sizes either temporarily or permanently but sacrifices image quality. Both distortion and artefacts occur when compression is introduced, hindering facial identification [90,125,129]. Keval and Sasse [90] found that the number of correct identifications of faces by untrained viewers decreased by 12–18% as MPEG-4 quality decreased and by 4–6% as Wavelet quality decreased (92–32 Kbps for both compression formats). They recommend a minimum of 52 Kbps video quality using MPEG-4 in order to achieve reliable and effective facial identification [90], albeit these results are for untrained practitioners and lower qualities would likely be reliable for trained FFC practitioners as well, perhaps not at the same magnitude. Vitek et al. [125] found correct identifications decreased from 88 to 48% as HEVC encoding quality decreased (30 kbps–15 kpbs) and they recommend 20 Kbps as a minimum threshold value. Compression employed in CCTV systems is lossy and, once performed during recording, cannot be removed or reversed. The types of distortion seen are pixelation, basis patterns, ringing, and blurring [99,130]. Recent advancements have been made improving FRT performance in light of compression artefacts; however, these artefacts remain a primary concern and drastically reduce accuracy and reliability [129]. An overview of the above-discussed various limiting factors of CCTV data in the application of MA and their specific effects in the process of facial comparison is presented in Table 2.

In consideration of our results, only two particular CCTV camera specifications under limited conditions and installation variations were tested [21,48,49]. However, there is a large number of manufacturers that produce CCTV equipment with different specifications, requirements, and support. Testing the extent to which various market standard CCTV cameras can affect facial comparison would be an ideal goal to strive towards. However, before attempting such a level of fine-tuning of facial comparison practice and requirements, broader aspects should be investigated. These would include investigating the contributions of each of the various aspects that appeared to contribute to a decrease in MA performance, particularly in an attempt to determine empirical thresholds for suitable image quality across various specifications and not only image resolution. Therefore, the common factors described above that affect quality should be investigated. For instance, developing a thorough understanding of distance-related distortion effects on MA between images from CCTV cameras and photographs could generate awareness of which features are altered more notably, and hence, increase inaccuracy at unfavorable distances. This is an important consideration for future work due to the varied conditions most CCTV systems are installed under and tailored to. The alternative of comparing faces captured under the exact same conditions would likely be more effective; however, it may not be feasible or cost-effective outside of an experimental scenario. In addition, the time discrepancy between a first set of images from a CCTV recording and a recapture for analysis may introduce further limitations on the equipment and conditions of image capture (e.g., different lighting, damaged camera, etc.). In addition, studying the precise effect of camera angle of incidence on MA in isolation would also contribute to improving its application. Clear thresholds for determining the angle steepness that significantly inhibits facial comparison will aid in screening the utility of current image data and to guide future surveillance system installation planning. Incorporating the average head tilt in these investigations would further contribute to perfecting these standards beyond the experimental scenario.

Actual digital image quality and minimum resolution allowing for facial comparison to take place should also be investigated. Based on this study’s conclusions with regard to low-resolution analogue CCTV, further investigations are needed in order to define a clear, quantifiable lower-end threshold that permits analysis. However, based on the actual accuracies and the approximate sizes of faces in each CCTV setting, it would appear that when a face is composed of approximately 18 × 26 pixels or less, such as the analogue CCTV setting employed in our study [48], FFC analysis would be severely compromised. This is a suggested preliminary lower threshold as despite conditions being mostly similar between standard CCTV and analogue CCTV, in terms of angle of incidence and SCD, faces in the standard CCTV were composed of approximately double the number of pixels (41 × 52 pixels) and a much higher accuracy and reliability were obtained [21]. This threshold remains well below Vitek et al.’s [125] and Utochkin’s [126] recommendations (minimum of 83 pixels for unknown faces). Developing clear, experimentally tested lowest acceptable quality thresholds, particularly under different settings and conditions, will aid both the surveillance industry and the forensic analysts conducting analyses. A useful consideration for future studies investigating all aspects of image quality in facial comparison would be to use an image quality scoring system. An example of such a scale was presented by Schüler and Obertová [22]. Implementing this scale in conjunction with the FISWG feature list for MA could aid in identifying a threshold of confidence for the analysis process based on image quality.

Despite these uncertainties, from our earlier results, we recommend that CCTV system installations transition towards the use of high-definition cameras installed at eye-level heights. However, this would limit the cost-effectiveness of CCTV installations, as one camera would have a more limited field of view at the lower height [99]. As such, more cameras would need to be installed to cover areas previously covered by a single or pair of cameras [99]. Installing eye-level IP CCTV cameras would invariably place these systems at higher risk of vandalism and sabotage; however, the authors think this risk and increased cost are worthwhile in the context of facial comparison analyses, considering the significantly higher accuracy obtained when comparing faces recorded on these types of installations. Angle of incidence close to zero, allowing for more closely matching face views, in conjunction with high-resolution footage and the resulting quality of the facial image (at a minimum representation of a face being 41 × 52 pixels) are ideal for FFC application. While no clear benefit or shortfall of color recordings were isolated, based on the qualitative assessment of the analyses conducted, the authors would recommend the inclusion of color CCTV to allow for a wider range of feature list applications, such as the inclusion of color-based features in the FISWG feature list. However, we would also recommend the removal of color-based features as discrepancies in lighting are common between realistic recordings and ideal photographs captured for comparison. The resulting analysis of facial feature descriptors relying on color, or other factors that can vary easily and unknowingly, such as luminescence, in response to slight variations in lighting conditions should be reconsidered or removed from feature lists, as they were found either unreliable or unusable in most comparisons.

### 4.2. Feature List Usage, Disguises, and Training

Both the FISWG and the ENFSI recommend MA as the best practice for forensic facial identification [27,125]. In addition, FISWG advises against the use of photo-anthropometry for facial image comparison and recommends superimposition only to be utilized in conjunction with MA [27]. FISWG developed and made freely available an extensive facial feature list for use in MA [51,54]. This list includes 18 facial components, each with associated descriptors, as well as a nineteenth descriptive component for use with uncategorized features [51,54]. The FISWG feature list is also the most exhaustive list available, including over 130 facial component characteristics and over 290 characteristic descriptors [51].

The application of facial feature lists, such as the FISWG one, is different from previous feature-based comparison methods that involved facial feature classification schemes. These classification schemes were used as a way for an analyst to score each facial feature into categories based on descriptive qualities (e.g., pointed chin, broad nose bridge, etc.) [59]. The FISWG approach instead expects a facial analyst to subjectively describe the compared faces by providing an extensive list of features and descriptors to use in order to make statements based on similarities and dissimilarities [27,51]. This descriptive approach is preferred as classification schemes are viewed as prone to high inter-observer error [55,59,109]. In addition, for classification schemes to be effective, they need to be tailored to specific populations, which has only been considered by two studies to date [58,127]. Population homogeneity, however, can be problematic for classification schemes, since high prevalence of a feature classification in a given population could result in an overlapping score, leading to erroneous false positive matches [60]. On the other hand, classification schemes may be too restrictive and make scoring near impossible under certain circumstances [26]. In this series of studies, the FISWG feature list was found to greatly aid both in the training of the analyst as well as during the analysis process to achieve mostly high accuracies and good reliability levels with the exception of the lowest quality of CCTV recordings. The feature list was also found to be applicable to African male faces due to its descriptive nature, as opposed to population-specific classification schemes. Certain descriptors were found cumbersome to utilize; for example, as mentioned above, skin color and luminance were often ignored due to a mismatch, despite confirmation that two faces were indeed the same. A revision of some of these descriptors would be required to optimize the analysis process and applicability of the FISWG feature list to a broader number of settings and CCTV conditions.

In addition, while the importance of a feature list in MA is undeniable, combining a systematic approach and a feature list must involve the option to discard potential dissimilarities when it is justifiable to do so. This is possible with large feature lists such as the FISWG one, which allows for exclusion of questionable or hard to analyze features. A smaller feature list be employed would compromise the exclusion of dissimilar features that could be justified as dissimilar due to image conditions, leading to the false exclusion of a positive face match due to features varying under the different image conditions. To this end, a threshold of the number of minimum features required to conduct an analysis should be investigated as neither the feature list [47] nor the concluding statements [17] provided one.

A further consideration to improve the applicability of the FISWG and any other feature lists would be to develop specific criteria to be applied for comparisons under different disguised or obstructed faces. Once established, these criteria could be included in analyst training to prioritize features by type of disguise. This approach would be applicable in settings where facial features may not be visible due to data loss or any other physical obstructions. This could prove particularly useful as the forms of “acceptable disguises” change throughout time—for example the use of face masks currently due to the spread of COVID-19. Face masks, which can vary in shape, size, and the resulting proportion of the face covered, have been shown to reduce automated facial recognition performance by 5 to 50% depending on the specific algorithm and extent of the face covered [76]. The deleterious effect in performance seemed to vary based on the color and shape of the masks as well [76]. It would, hence, be crucial to consider face masks in further tests of MA under disguised conditions as their impact on human observer-based facial comparison has not yet been considered.

**Figure 3:**
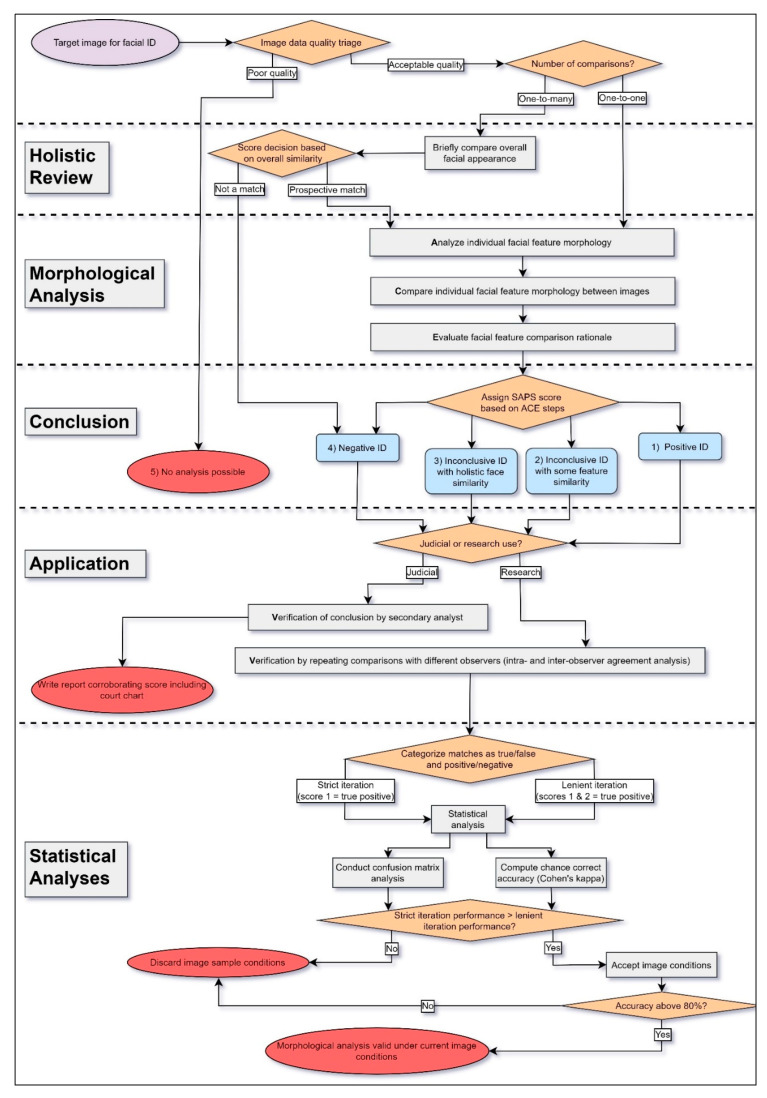
Flow diagram of the recommended morphological analysis process. This approach to morphological analysis uses an ACE-V method in conjunction with the FISWG feature list [47], with the inclusion of the ENFSI’s image quality triaging [114] and the use of the South African Police Services (SAPS) scoring criteria [17] as adapted for research application [21]. Statistical analyses for research use are also recommended based on our recent work [48] to allow for more detailed result interpretation and comparison among future studies.

While the current studies validated MA when the FISWG guidelines [27,47] were used, an array of further studies is required. To continue this work, the Wits Face Database will need to be updated and expanded to include many more possible permutations of analysis, including female individuals, cosmetic make-up treatments, and face mask disguises. Future studies should also attempt to quantify the acceptable loss of facial feature information in order to successfully compare faces across a multitude of possible situations. Once that goal is achieved, better guidelines and practice frameworks can be created for legal procedures involving FFC by MA. Our recommended method of conducting MA is shown in Figure 3. This approach outlines the stepwise process of applying the recommended image quality triage by the ENFSI [114] to FISWG’s ACE-V application [27] of the FISWG feature list [47] and then subdivided by intended use in a research or judicial context. Based on the application context, different approaches are used for verification; in addition, research use of MA would require further statistical analyses (Figure 3).

Based on the outcomes of our group’s studies, expanding current training programs and developing new ones to increase the competence of facial comparison experts will be crucial for consistent and reliable application of FFC. The application of a feature list and an ACE-V approach by members of the public in forensic facial comparisons is not sufficient to achieve expertise. Training experts with the explicit role to conduct FFC analyses, with the use of the FISWG feature list and an ACE-V approach, is of utmost importance in a judicial context [131]. The role of expertise is particularly relevant in FFC, since unfamiliar face matching is considered complex and unreliable on all accounts [97,122]. Experts, in fact, perform notably better than members of the public [42], even when image quality was taken into consideration [132]. This expertise undoubtably arises from training in the nuances of faces, such as facial expressions and ageing changes, and acceptable anatomical variations and image-based variations between faces, beyond just the inclusion of the use of a feature list. The need for adequate training of all FFC practitioners is vital to the good standing of the practice and its admissibility in a legal context. Particularly when considering that certain countries may experience a shortage of expertise and heavy caseloads, such as South Africa, where only 30 trained specialists in the entirety of the national police force are trained to conduct FFC and testify in court to defend their conclusions [17].

The FISWG has put forward a document describing guidelines for training and expertise requirements of FFC analysts and trainers [133]. While these guidelines are crucial to the development of training courses, to the authors’ knowledge, no formal standardized training or certification platforms exist for FFC [17]. A recent study on the performance of informal training courses on facial comparison suggested that there are large discrepancies between courses in improvement of facial examiner expertise [134]. We hope our recommended stepwise process to the applications of MA (Figure 3) will aid in streamlining both MA training and application. Recently, members of our research group proposed an outline for a training course with a three-tiered approach offered to the police force [17]; it inadvertently follows most of the proposed guidelines from FISWG. The first tier of training involves developing basic background knowledge of facial anatomy, evidence evaluation, image science, facial recognition psychology, and court proceedings, among other topics [17]. The second tier involves training in detailed MA using the FISWG standards and developing court-ready reports and charts [17]. The third tier is a national specific tier that involves advanced training in court proceedings and evidence presentation as well as troubleshooting from past casework in order to also train experienced peer reviewers [17], who are vital to the ACE-V application of MA. While this approach to training has not been experimentally tested, the trained police members have found success in their roles as facial examiners.

## 5. Conclusions

The outcomes and recommendations arising from these studies should be considered under the limitations of the investigative approach deployed. These studies attempted to simulate real-world conditions, with its array of limitations, in a select number of scenarios. These scenarios included ideal comparable photographic images, standard digital CCTV, eye-level installation digital CCTV, standard monochrome analogue CCTV, and two common disguises—sunglasses and brimmed caps. The varied circumstances of facial data were pre-set under certain conditions to attempt some level of standardization required for experimentation. In doing so, although realistic, the conditions were limited to the major questions broadly investigated by each study [21,48,49]. Although these broad categories were considered as realistic examples of CCTV image quality and conditions, there are multiple factors that influence the quality of an image for facial identification. Image quality relies on more than just equipment resolution capacity; it involves lighting conditions, angle of incidence, SCD, distortions, color, visibility of features, and more. In testing the specific conditions outlined in each of the above studies, controlling for or identifying which of the multiple limiting factors contributed to the poor performance of MA would be impossible. Even when within likely tolerable degrees, these limiting factors cannot be isolated from one another in certain circumstances. However, with this baseline of conditions and considerations, future studies can be tailored to the specific limitations that CCTV imposes on FFC in a highly controlled setting to determine the exact contribution of each of these limiting factors to the accuracy of MA.

With these concerns and limitations clearly stated, future studies should be focused to target specific limiting factors individually in order to develop a clear threshold for image data to be usable for facial comparison. While other approaches to facial identification as a whole may also be gaining popularity, such as the increasing performance of automated systems [31,75,76] and the deployment of super-recognizers [135,136], continued research in forensic facial comparison by MA is crucial as the most universally applicable and reliable method. The importance of MA-based FFC is especially noteworthy in law enforcement applications, where the majority of available image data is of low to poor quality [113]. As such, the authors strongly advise that trained human observer-based MA, using the FISWG feature list [47] and an ACE-V approach [27], should remain the principal method of facial comparison for identification purposes, as recommended by both the FISWG and ENFSI [27,114].

## Figures and Tables

**Table 1:** Composition of the Wits Face Database [41] CCTV data and detailed data loss experienced during database development as a result of the CCTV systems’ technical limitations.

**Database Cohort Organization**	**Unique Individuals**	**Photographs**	**Corresponding CCTV ^1^ Recordings**	**Data Loss (%)**
ST ^2^ CCTV ^1^—ST ^2^ Photographs	98	980	89	9.2%
Eye-level CCTV ^1^—ST ^2^ Photographs	108	1080	76	29.6%
ST ^2^ CCTV ^1^ with Cap—ST ^2^ Photographs	45	450	34	24.4%
ST ^2^ CCTV ^1^ with Cap—ST ^2^ Photographs	41	410	31	24.4%
Total IP ^3^ CCTV ^1^ Data	292	2920	230	21.2%
Analogue CCTV ^1^—ST ^2^ Photographs	111	1110	107	3.6%
CCTV ^1^ Grand Totals	403	4030	337	16.4%

^1^ CCTV = closed-circuit television; ^2^ ST = standard; ^3^ IP = internet protocol.

**Table 2:** Summary of CCTV systems’ technical limitations in the application of morphological analysis.

General Limitations	Specific Limitations	Effects
Camera placement	Camera height above ground [21,48,49,99,100] Angle of incidence [21,48,49,100] Subject-to-camera distance [103,104,127]	Image composition affected—target size and screen/picture height [21,48,99] Reduction of observable facial features [21,48,49,100] Perspective distortion [103,104,127]
Camera specifications	Analogue or digital [82,115,116] Sensor size [99] Pixel count [48,99] Lens focal length [99]	Reduced image quality [21,48,99] Image distortion and artefacts [99]
Lighting conditions	Ambient lighting [99,107,108] Infrared vision [93,107,108]	Loss of facial detail [48,49] Shadows and overexposure form artificial boundaries and altered facial appearance [49,106] Optical distortions [99]
Image quality	Resolution [21,48] Pixelation [48,92] Noise/grain [99] Video compression [90] Color [48]	Low clarity [99,103] Reduced useable detail [21,48,82,90,92] Face matching ability reduced [21,48,90,125,129]
Data loss and corruption	Network infrastructure [41,72] Software [99] Hardware [41,99] Imminent weather [41] Power outages [41] Compression rate [90] Anti-forensic techniques [109,110,111,112]	Inconsistent network connection and coverage—transfer corruption [41] Partial or complete data loss [41] Data tampering and removal [109,110,111,112]

## Data Availability

The facial image data used in Figure 1 and Figure 2 for the current paper are of the corresponding author and publicly available as part of the sample images of the Wits Face Database [41], reproducible under an open access license distributed under the terms of the Creative Commons Attribution License. This license permits unrestricted use, distribution, and reproduction in any medium, provided the original work is properly cited. These images can be accessed as part of the Wits Face Database data note, via the supplementary material for the Wits Face Database [41].
